# Improving Patient Access to Musculoskeletal Treatments and Medical Student Musculoskeletal Education via Free Clinic Services

**DOI:** 10.7759/cureus.47996

**Published:** 2023-10-30

**Authors:** Rock P Vomer, Rayghan S Larick, Abigail Bent, Chris Fungwe, Parth S Contractor, Emma York

**Affiliations:** 1 Family Medicine, Avance Care, Raleigh, USA; 2 Family Medicine Research, Mayo Clinic - Jacksonville, Jacksonville, USA; 3 Physical Medicine and Rehabilitation, Eastern Virginia Medical School, Norfolk, USA; 4 Family and Community Medicine, Eastern Virginia Medical School, Norfolk, USA; 5 Family Medicine, Eastern Virginia Medical School, Norfolk, USA; 6 Family and Preventive Medicine, Prisma Health University of South Carolina, Columbia, USA

**Keywords:** student education, osteopathic technique, orthopaedics and sports physical therapy, underserved populations, underserved clinics

## Abstract

Introduction

Homeless patients are at higher risk for musculoskeletal conditions but have limited access to treatments. Physical therapy (PT) and osteopathic manual therapy (OMT) are treatments for acute and chronic musculoskeletal conditions. Here, we outline establishing a free specialty clinic to provide PT and OMT to underserved patients.

Methods

At Eastern Virginia Medical School’s Health Outreach Partnership of EVMS Students (HOPES) free clinic, we established a volunteer student, resident, and attending-led specialty clinic to provide exercise therapy, manual therapy, and injections to underserved patients.

Results

Student volunteering resulted in significant improvement in student confidence with musculoskeletal physical exam skills and their ability to diagnose musculoskeletal conditions. Patients of the clinic reported significant improvement in pain and mobility.

Discussion

This clinic is a novel activity that improves student musculoskeletal medical education and patient access to musculoskeletal condition treatments. Exposing students to PT and OMT services increased student awareness of the PT scope of practice, OMT's use as a component for patient treatment increased confidence in the appropriate applications of OMT for patient care. Implementing a free PT and OMT clinic at an established free clinic can improve musculoskeletal medical education and enhance patient care for underserved populations.

## Introduction

In the United States, musculoskeletal disorders affect an estimated 126.6 million adults annually, resulting in $874 billion in treatment costs, lost wages, and healthcare visits [1]. Musculoskeletal disorders represent more than 50% of disabling conditions, are a primary reason for healthcare visits, and are a total of 20% of emergency departments and primary care clinic visits [2]. Homeless people are at an even higher risk of musculoskeletal conditions but face limited access to treatment and frequently seek care in emergency departments.

Physical therapy (PT) is used for both acute and chronic musculoskeletal conditions and can reduce patient treatment costs by up to 72% [3]. Treatment courses require 5-12 sessions to achieve results [4]. For the homeless population, PT services are difficult to obtain for various reasons: lack of insurance or finances, transportation difficulties, difficulty performing home exercise programs due to housing situations or lack of home equipment, and others. This leaves an already vulnerable patient population without a valuable treatment option.

In primary care, musculoskeletal complaints are the second leading cause of patient visits and account for 30%-40% of primary care visits [2,5,6]. In 2021, 16.3% of medical graduates entered primary care training programs [7]. Although musculoskeletal condition caseloads remain high among these specialties, musculoskeletal education in medical school remains notoriously low: when medical students' application of musculoskeletal knowledge has been tested, 81% of students failed because of the inadequacy of the curricula [8].

Osteopathic manipulative therapy (OMT), including spinal manipulation, is a recommended first-line treatment modality for low back pain [9,10]. OMT is a skill in which doctor of osteopathic medicine (DO) students are trained during medical school, where they receive 300-400 hours of combined didactic and hands-on skills training. DOs accounted for 11% of the physician workforce and contributed 7,500 graduates in 2021. Manual therapy, an umbrella term for various types of hands-on manipulations that include OMT, is a skill set used by many healthcare professionals, including physicians, physical therapists, athletic trainers, and chiropractors. Although DOs commonly use OMT, MDs who are trained in OMT can also perform this skill. When a comparison of MD and DO students evaluated the perceived adequacy of their musculoskeletal education, DO students reported much higher perceived adequacy than did their MD counterparts (57% vs 26.8%, respectively); in addition, DO students reported feeling more adequately prepared for standardized exams due to their medical education than did their MD counterparts (36.6% vs 8.1%, respectively) [10,11].

Several blinded randomized controlled trials that studied the effect of OMT on low back pain indicated that OMT significantly reduced low back pain [12]. Further, OMT for low back pain resulted in 18.5% fewer prescriptions written, 74.2% fewer X-rays ordered, 76.9% fewer referrals to other providers, and 90% fewer magnetic resonance imaging scans ordered [13]. OMT, which can be performed in-office during an initial visit for a musculoskeletal complaint, is a useful tool to help decrease patient pain and increase patient function [14].

Physical exam skills include a variety of special tests for musculoskeletal conditions that have varying degrees of sensitivity and specificity, as do any laboratory values or test results. Physical examination skills facilitate the correct diagnosis of musculoskeletal conditions and can be performed at the bedside during the initial phase of a patient evaluation [15]. Both PT and OMT rely on the provider's understanding of the musculoskeletal system and musculoskeletal condition diagnosis, allowing bedside diagnoses during history taking and physical exams, often without the need for imaging studies. Physicians who are well-trained in musculoskeletal conditions likely are better equipped to diagnose a variety of musculoskeletal conditions with and without imaging and to provide robust treatment plans to patients.

## Materials and methods

To begin the clinic at the Eastern Virginia Medical School's (EVMS) Health Outreach Partnership of EVMS Students (HOPES) free clinic, we first developed our standardized operating procedures (SOP) and identified an appropriate location for the clinic at HOPES. The SOP specifically outlined how the clinic would run and outlined which services patients were offered and the needed supplies. The PT AND OMT clinic was held every other week, allowing for both two- and four-week patient follow-ups. For each clinic, we scheduled eight patients. Each appointment was one hour and included diagnosis and treatment. A clinic flow timeline was provided to each student and clinician to assist with time management. In total, the clinic ran for approximately 2.5 hours. HOPES is located at the Norfolk Health Department and includes a large open space similar to that of a PT office, including space for four exam tables where patients can be evaluated and receive treatments. This was a quality improvement project aimed focused on improving student knowledge and did not require ethical approval by the EVMS IRB.

The clinic is run by volunteer students, residents, and attendees. Four student clinic coordinators were present at each clinic and helped with scheduling and notifying patients of upcoming appointments, ensuring we had enough student volunteers for the clinic, checking patients in, and ordering clinic supplies. Our resident leaders helped with clinic design and patient treatment plan creation; they also were present for all clinics to oversee and perform procedures. Attendees were present to oversee clinic operations and procedures.

Each clinic followed the same format from start to finish. Once scheduled patients arrive, they complete paperwork per their musculoskeletal complaint: Ankle Joint Functional Assessment Tool, American Shoulder and Elbow Surgeons-Elbow Scoring System, Oswestry Low Back Pain Disability Questionnaire, Foot and Ankle Ability Measure, Hip Outcome Score, Shoulder Pain and Disability Index, and the Western Ontario and McMaster Universities Osteoarthritis Index. The patient-reported outcome measures, and visual analog scores were used to track patient progress and response to treatment each session. With each visit, a new form is filled out to allow for symptom monitoring and function improvement. Next, the clinic coordinators bring the patient back to the specialty clinic area, where students collect the patient's history and perform a physical exam. Students are provided with a history outline and approach to a physical exam to aid with efficiency. Afterward, students present patient cases and physical exam findings to the residents and/or physical therapist staff volunteering at that evening's clinic. Next, the resident/PT examines the patient along with the student team, asking for any additional information and performing any additional physical exam maneuvers necessary for accurate diagnosis and treatment plan formation.

Based on the physical exam findings, patients are led through a series of therapeutic exercises that become the patient's home exercise program. Home exercise program development is done on a case-by-case basis depending on the patient's functional status, physical exam findings, and musculoskeletal condition. Next, trigger point injections or joint injections, as appropriate, are administered. Stim modality is applied to the area being treated and, if appropriate, followed by OMT. Lastly, ice is applied to the area being treated. Following treatments, the patient is given a copy of their home exercise plan and any equipment necessary to perform exercises (TheraBand or towel). We used Hep2go (HEP2go, Inc., Maricopa, AZ) to build patient programs, which include the number of repetitions and sets for each exercise and can be printed in English or Spanish.

Before leaving, the patient was scheduled for a follow-up visit and filled out a satisfaction survey. Following the visit, students submit a subjective, objective, assessment and plan (SOAP) note to our Electronic Medical Record, allowing future students and clinicians to quickly refer to previous findings and treatments, assess patient outcomes and progress, and provide continuity of care. Following the clinic, student volunteers are emailed a student satisfaction survey.

The current clinic format allows students to practice collecting a history and performing a physical exam. Additionally, our clinic setup allows students who attend the OMT class we established at EVMS to utilize the skills they learned there during the treatment phase of our clinic. Due to the HOPES guidelines, only residents or attendings are allowed to perform procedures, including trigger point injections and joint injections. Satisfaction surveys for both students and patients facilitate continual assessment of clinic functions.

## Results

Student surveys made in Qualtrics (Qualtrics International Inc., Seattle, Washington) were sent via email at the conclusion of the clinic and were used to assess change in student knowledge and comfort with OMT, musculoskeletal examination, diagnosis, and treatment. This information was used to refine clinic processes and track student learning.

A total of 17 prospective participants clicked on the participation link. Six cases were removed due to 100% missing quantitative data, yielding a usable sample of 11. Among participants (n = 11), 77.4% (n = 7) were in the MD program, and 22.6% (n = 4) in the PA program. For student level of training, 32.2% (n = 3.5) were in their first year, 41.9% (n = 4.6) in second year, 16.1% (n = 1.76) in third year, and 6.5% (n = 0.66) in fourth year. Further, 37.8% (n = 4.15) of participants had heard of OMT prior to the OMT/PT clinic, and 61.3% (n = 6.74) had not.

Internal consistency reliability (Cronbach’s coefficient alpha (α) and McDonald's coefficient omega (ω)) was conducted for both pre- and posttest scores. Results revealed acceptable internal consistency reliability evidence of scores for the pretest (α = 0.78, ω = 0.77) and posttest (α = 0.73, ω = 0.74). A review of skewness and kurtosis values indicated that the pretest and posttest scores were consistent with a normal distribution (skewness < 2.0 and kurtosis < 7.0).

**Figure 1:**
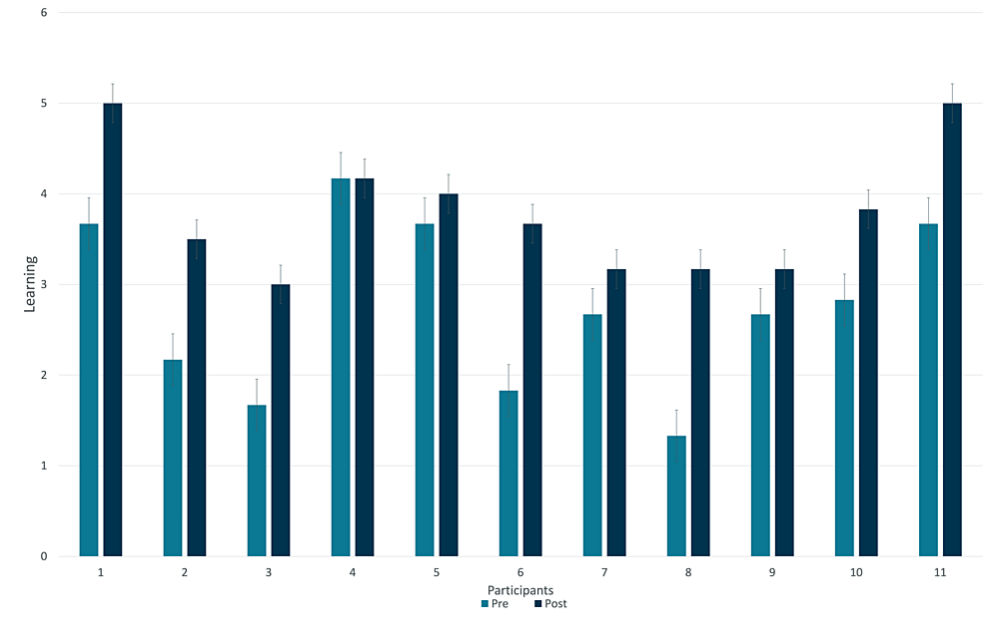
Change in Student Knowledge Before and After Participation in the Clinic This graph demonstrates the change in student knowledge before and after participating in the OMT clinic. The learning scale was based on the total number of questions on the knowledge quiz. The lowest score was 0, and the highest score was 6.

**Figure 2:**
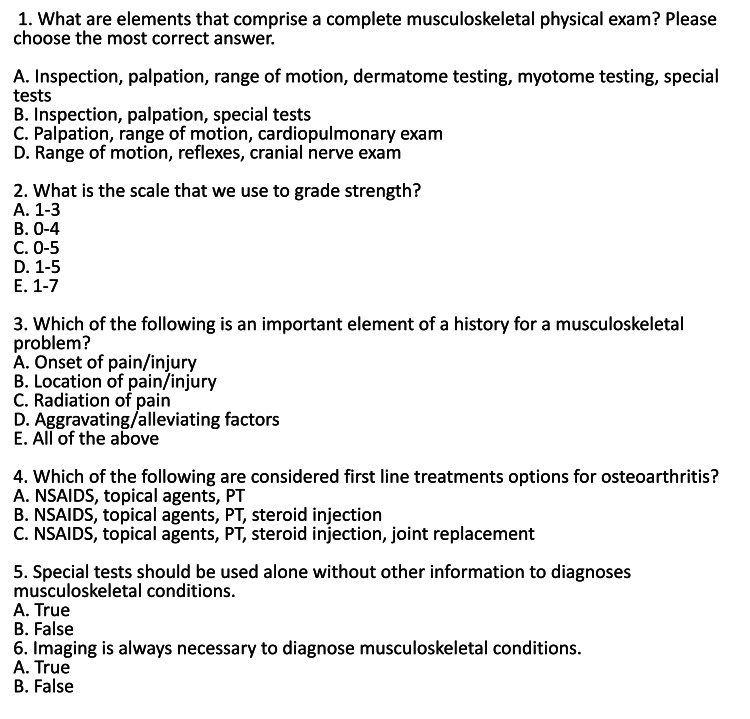
PT/OMT Clinic Knowledge Survey This survey was utilized to assess the change in student learning before and after participation in the clinic. *NSAID = Nonsteroidal Anti-inflamatory Drug, OMT = Osteopathic Manipulative Treatment, PT = Physical Therapy.

Time was the independent variable (IV) and included the following two levels: 1 = preintervention and 2 = postintervention. Results revealed a statistically significant increase in participants' musculoskeletal learning between the pretest (M = 2.76, SD = 0.94) and posttest (M = 3.79, SD = 0.71), 95% CI = -1.44, -.62, t(10) = -5.57, p < 0.001, d = 1.24 (Figure 1). The student knowledge was assessed using a simple questionnaire (Figure 2).

**Figure 3:**
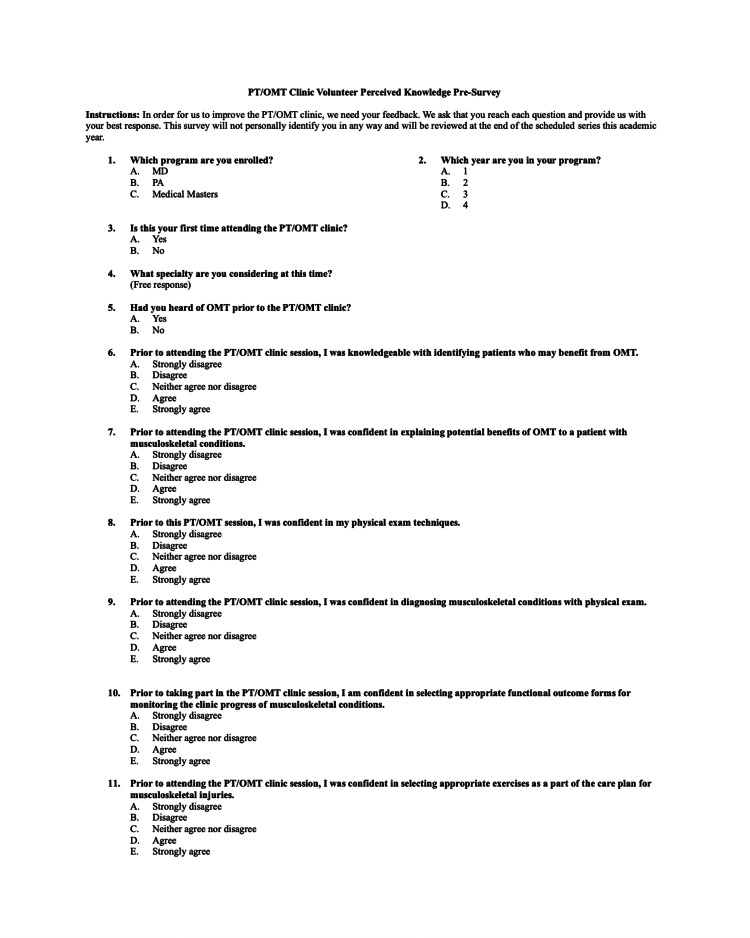
PT/OMT Clinic Volunteer Perceived Knowledge Pretest Survey This survey was utilized to assess student confidence with OMT prior to participation in our clinic.

**Figure 4:**
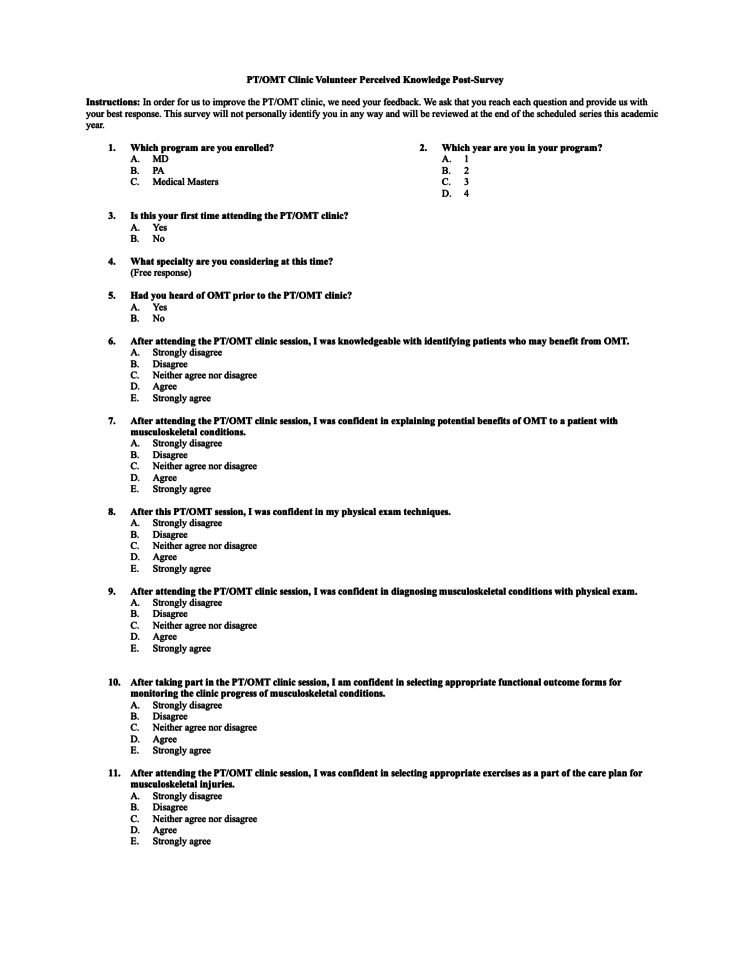
PT/OMT Clinic Volunteer Perceived Knowledge Posttest Survey This survey was utilized to assess student confidence with OMT after participation in our clinic.

The dependent variable (DV) was participants' total, continuous-level score on the OMT confidence survey. A dependent samples t-test revealed a statistically significant increase in participants' OMT confidence between the pretest (M = 13.57, SD = 4.72) and the posttest (M = 21.53, SD = 3.65), t(29) = 9.68, p < 0.001, d = 4.50 (Table 1). The results revealed that participation in the OMT clinic was associated with statistically significant increases in participants' OMT confidence. The practical significance (effect size) of the results was in the strong range based on Mvududu and Sink14 guidelines for interpreting Cohen’s d. The survey to assess student confidence is included in the Appendix (Figures 3-4).

**Table 1:** Student Perceived Confidence This table reflects student confidence with OMT before and after participating in our clinic. *OMT = Osteopathic Manipulative Treatment, PT = Physical Therapy

Which program are you enrolled:	Which year are you in your program:	Is this your first time attending the OMT/PT clinic?	What specialty are you considering at this time?	Had you heard of OMT prior to the OMT/PT clinic?	Prior to attending the OMT/PT clinic session, I was knowledgeable with identifying patients who may benefit from OMT.	After taking part in the OMT/PT clinic session, I feel knowledgeable with identifying patients who may benefit from OMT.	Prior to attending the OMT/PT clinic session I was confident with explaining potential benefits of OMT to a patient with musculoskeletal conditions.	After taking part in the OMT/PT clinic session, I am confident with explaining potential benefits of OMT to a patient with musculoskeletal conditions.	After taking part in the OMT/PT clinic session, I am confident with explaining potential benefits of OMT to a patient with musculoskeletal conditions.	Prior to attending the OMT/PT clinic session, I was confident with physical exam techniques.	After taking part in the OMT/PT clinic session, I am confident with physical exam techniques.	Prior to attending the OMT/PT clinic session, I was confident in diagnosing musculoskeletal conditions with physical exam.	After taking part in the OMT/PT clinic session, I am confident in diagnosing musculoskeletal conditions with physical exam.	Prior to taking part in the OMT/PT clinic session, I am confident in selecting appropriate functional outcome forms for monitoring the clinic progress of musculoskeletal conditions.	After taking part in the OMT/PT clinic session, I am confident in selecting appropriate functional outcome forms for monitoring the clinic progress of musculoskeletal conditions.	Prior to attending the OMT/PT clinic session, I was confident in selecting appropriate exercises as a part of the care plan for musculoskeletal injuries.	After attending the OMT/PT clinic session, I was confident in selecting appropriate exercises as a part of the care plan for musculoskeletal injuries.
PA	3	No	Women’s health, dermatology, primary care, surgery	Yes	Neither agree nor disagree	Strongly agree	Agree	Strongly agree	Strongly agree	Strongly agree	Strongly agree	Agree	Strongly agree	Neither agree nor disagree	Strongly agree	Neither agree nor disagree	Strongly agree
MD	3	Yes	Cardiology	No	Disagree	Agree	Strongly Disagree	Neither agree nor disagree	Neither agree nor disagree	Neither agree nor disagree	Neither agree nor disagree	Disagree	Neither agree nor disagree	Neither agree nor disagree	Agree	Disagree	Agree
MD	2	No	EM	No	Strongly Disagree	Agree	Strongly disagree	Neither agree nor disagree	Neither agree nor disagree	Neither agree nor disagree	Agree	Disagree	Neither agree nor disagree	Disagree	Disagree	Strongly disagree	Disagree
MD	4	No	PM&R	Yes	Strongly agree	Strongly agree	Agree	Agree	Neither agree nor disagree	Agree	Agree	Agree	Agree	Agree	Agree	Agree	Agree
MD	4	Yes	EM	Yes	Strongly agree	Strongly agree	Neither agree nor disagree	Agree		Neither agree nor disagree	Neither agree nor disagree	Neither agree nor disagree	Agree	Agree	Agree	Agree	Agree
MD	1	Yes	Orthopedics	No	Disagree	Agree	Strongly disagree	Neither agree nor disagree	Neither agree nor disagree	Disagree	Strongly agree	Disagree	Neither agree nor disagree	Strongly Disagree	Neither agree nor disagree	Neither agree nor disagree	Agree
MD	2	No	PM&R, internal medicine	No	Neither agree nor disagree	Agree	Agree	Disagree	Disagree	Disagree	Neither agree nor disagree	Disagree	Disagree	Disagree	Agree	Neither agree nor disagree	Agree
MD	1	Yes	Family medicine	Yes	Disagree	Agree	Disagree	Agree	Agree	Strongly disagree	Neither agree nor disagree	Strongly Disagree	Neither agree nor disagree	Strongly Disagree	Disagree	Strongly disagree	Neither agree nor disagree
MD	1	Yes	Orthopedics, general surgery	Yes	Agree	Agree	Agree	Agree	Agree	Strongly disagree	Disagree	Disagree	Neither agree nor disagree	Agree	Agree	Strongly disagree	Disagree
PA	3	Yes	Critical care or emergency medicine	Yes	Neither agree nor disagree	Agree	Neither agree nor disagree	Agree	Strongly agree	Disagree	Agree	Agree	Agree	Neither agree nor disagree	Neither agree nor disagree	Disagree	Agree
PA	3	Yes	ER	Yes	Agree	Strongly agree	Agree	Strongly agree	Agree	Disagree	Strongly agree	Strongly agree	Strongly agree	Strongly agree	Strongly agree	Disagree	Strongly agree

## Discussion

This clinic represents a novel method of improving both student confidence in musculoskeletal medical topics and improving underserved patient access to treatments for musculoskeletal conditions. Overall, based on the findings, implementing a free PT and OMT clinic in association with a community free clinic significantly improves both student understanding of musculoskeletal conditions and OMT. In each clinic, students noted a significant increase in the amount of time they had to learn, practice, and understand physical exam skills in comparison to the time allocated during the musculoskeletal block in school. Overall, students noted a substantial improvement in comfort with a physical exam for musculoskeletal conditions and felt they developed a standard approach for physical exams. Additionally, they noted appreciation for OMT and its uses for their future patients and increased comfort with discussing OMT's uses with patients. At EVMS, students noted that their formal physical exam skills training consisted of one-hour sessions a few times per year. However, with the addition of this curriculum, their physical exam training was increased to two to four hours per month, depending on how many clinics they volunteered for.

The biggest limitations of the study were students completing the survey and having repeat student volunteers at the clinic. Additionally, for the patients, due to the socioeconomic factors surrounding this patient population, consistent follow-up was difficult to achieve. Thus, many of the patients whose pain improved were lost to follow up prior to being able to have them complete another survey, or those whose pain did not improve or worsened did not show up for other reasons.

Patients treated by the clinic noted an improvement in both pain and mobility. Every patient who was evaluated and treated by the clinic received a home exercise program, OMT, and either transcutaneous electrical nerve stimulation (TENS) unit application or ice. Additionally, these patients were able to receive joint injections and/or trigger point injections at no cost. Our patients were evaluated and treated multiple times over the course of a few months with little to no imaging or prescription medications required. Medications that were utilized were low-cost, over-the-counter options, including NSAIDs, acetaminophen, and diclofenac gel. The OMT/PT clinic served patients who typically have their pain attended in the emergency room and provided them with imaging and medications, along with free access to a multi-modality approach to pain and musculoskeletal conditions.

Consistently evaluating both patients' and students' experiences helped us continually refine our process. After a few months of visits, we established an effective clinic flow that both provided good patient care and student education opportunities. Based on feedback from both patients and students, we think this successfully addressed the problem of student education at EVMS for those who volunteered with us. Of course, not all students at EVMS volunteer at the clinic, so we were not able to affect medical education for all students. Additionally, not all underserved patients in our community are seen at the HOPES clinic, so this resource is not utilized by all homeless or uninsured patients in the community. However, we think this is a good start to expanding patient access to additional treatment modalities for musculoskeletal conditions.

We plan to continue the clinic on a bi-monthly basis to help expand patient access to OMT and PT services, as well as educate students at EVMS. Our goal is to continue to increase student knowledge in physical exam skills, musculoskeletal medicine, and OMT. In the future, we hope to increase the number of students who are involved in the clinic and increase the number of residents, attendings, and physical therapists available to oversee students at the clinic. Through the combined efforts of the class and free clinic, we have not only been able to educate students but we have also given them the ability to apply their skills. Most importantly, we have had the opportunity to improve patient access to care.

## Conclusions

Our clinic represents a novel method of improving student musculoskeletal medical education while increasing access to care for the underserved patient population for treatments of musculoskeletal conditions. Based on our findings, the implementation of a free PT and OMT clinic in association with a community free clinic significantly improves both student understanding of musculoskeletal conditions and OMT. In each clinic, students noted a significant increase in the amount of time they had to learn, practice, and understand physical exam skills in comparison to the time allocated during the musculoskeletal block in school. Further studies should be conducted to compare this clinical-based method of instruction versus traditional methods of teaching musculoskeletal conditions and OMT.
